# Does Cisapride, as a 5HT_**4**_ Receptor Agonist, Aggravate the Severity of TNBS-Induced Colitis in Rat?

**DOI:** 10.1155/2012/362536

**Published:** 2012-07-24

**Authors:** Azadeh Motavallian, Mohsen Minaiyan, Mohammad Rabbani, Parvin Mahzouni, Sasan Andalib, Alireza Abed, Mohammad Reza Babavalian

**Affiliations:** ^1^Isfahan Pharmaceutical Sciences Research Center, School of Pharmacy and Pharmaceutical Sciences, Isfahan University of Medical Sciences, Isfahan 8146-73461, Iran; ^2^Department of Pharmacology and Toxicology, Faculty of Pharmacy, Isfahan University of Medical Sciences, Isfahan 8146-73461, Iran; ^3^Department of Clinical Pathology, School of Medicine, Isfahan University of Medical Sciences, Isfahan 8146-73461, Iran; ^4^Neurosciences Research Center, Tabriz University of Medical Sciences, Tabriz 8146-73461, Iran; ^5^Islamic Azad University, Qazvin Branch, Qazvin 8146-73461, Iran

## Abstract

There is a pressing need for research that will lead to the reveal of targets designed to analyse the possible pathways for the treatment of IBD. Because of the probable involvement of serotonin in inflammatory conditions of intestine and the important role of 5HT_4_ receptors in GI function, the investigation of the role of 5HT_4_ receptors in the pathogenesis of IBD will be interesting. The aim of this study was to investigate the effects of cisapride, a 5HT_4_ receptor agonist, in trinitrobenzenesulfonic-acid-(TNBS) induced rat colitis. Two hours subsequent to induction of colitis using TNBS in rats, cisapride (2 mg/kg, intraperitoneally (i.p); 4 mg/kg, orally (p.o)) and dexamethasone (1 mg/kg, i.p; 2 mg/kg, p.o) were administrated for 6 days. Animals were thereafter euthanized; macroscopic, histological, and biochemical assessments and ELISA test were carried out on distal colon samples. Our data showed that dexamethasone treatment (i.p, p.o) significantly decreased macroscopic and microscopic damage and also biochemical markers, but there were no significant differences in aforementioned parameters between cisapride (i.p or p.o) and TNBS-treated rats. It can be deduced that because the severity of colitis produced by TNBS is massive (through various pathways), cisapride could not bring about more colitis damages through 5HT_4_ receptors. Based on the present study further researches are required for investigating the exact roles of 5HT_4_ receptors in the pathogenesis of ulcerative colitis.

## 1. Introduction

Inflammatory bowel disease (IBD), comprising ulcerative colitis (UC) and Crohn's disease (CD), is a chronic inflammatory disorder of the gastrointestinal  (GI)  tract characterized by a relapsing course [1]. Despite extensive research in the last decades, the etiology of IBD and the initial event of the inflammatory cascade still remain ambiguous [2]. In fact, genetic, immune, and environmental factors are involved in its pathogenesis [2, 3]. IBD is characterized by massive cellular infiltrates and pertains to abnormalities of the immune system involving heightened number of CD4^+^ T lymphocytes, mast cells, neutrophils, and eosinophils [4]. These conditions bring about inflammation, ulceration, edema, diarrhea accompanied by blood and/or mucus, fever, abdominal pain and gastric dysmotility, anemia, weight loss, and a range of extraintestinal symptoms [5]. Many medical therapies have been proposed for IBD like salicylates, glucocorticoids, and immunosuppressives; nonetheless, available medicines are not universally effective and result in marked deleterious effects, and therefore the medical management of IBD remains challenging, and investigations on novel treatments are required [6]. 

5-hydroxytryptamine (5-HT, serotonin) is an important gastrointestinal  (GI)  signaling molecule involved in motor, secretory, and sensory functions. It is also found in the immune-inflammatory axis and affects the mammalian immune response [7, 8]. These actions are mediated by a large family of serotonin receptors located within the neural circuitry and on a variety of other cell types in the gut [9]. Of the 5-HT receptors expressed in the intestines, the 5-HT_3_ and 5HT_4_ receptors have been the most widely studied in regards to GI function [10]. Because of extensive distribution of 5HT_3_ and 5HT_4_ receptors in intestine and especially in colon, more recently interest is focusing on the roles of these two receptors as pharmacotherapeutic targets for the treatment of GI disorders [10]. In this regard 5-HT_3_ receptor antagonists are used to treat nausea and emesis associated with chemotherapy [11] and for functional disorders associated with diarrhea [12]. In addition, recent investigations have revealed new clinical indications for this class of drugs. Preliminary data have shown the analgesic effects of ondansetron and tropisetron in patients with neuropathic pain and chronic inflammatory joint diseases, respectively [13, 14]. 

Animal models of IBD have been central to the investigation of the pathophysiology of the disease and are valuable tools for drug testing and development. The murine model of TNBS-induced acute or chronic colitis in rat is one of the most widely used models in both pharmacological and pathophysiological studies [15]. UC is said to be associated with the changes in EC cell numbers, 5-HT content, and enhancement of serotonin secretion. Additionally, an augmentation in the amount of 5-HT and secretion resembling the pattern of UC was seen in TNBS model of colitis [16]. 

In spite of the fact that the highest expression of 5HT_4_ receptors is in the distal colon [17], little is known about the role of this receptor in pathogenesis of GI diseases such as ulcerative colitis. Nowadays, 5-HT_4_ receptor agonists are used as promotility agents to promote gastric emptying and to alleviate constipation. Cisapride, as a 5HT_4_ receptor agonist, was developed with the intention of treating both upper and lower gastrointestinal dysfunction (in particular, for gastroesophageal reflux disease and dyspepsia), but finally it has been removed from the United States market secondary to the risk of arrhythmias in those with predisposing conditions [10]. In addition, Hoffman et al. demonstrated the 5-HT_4_ receptor expression by EC cells, goblet cells, and enterocytes [17]. Therefore the stimulation of serotonin secretion by any or all of these cell types through 5HT_4_ receptors could promote the cascades which 5HT is involved in them. Intestinal inflammation may arise from a change in 5-HT-producing enterochromaffin (EC) cells and an increase in 5-HT content associated with the pattern of IBD [18]. It is also worth noting that serotoninergic receptors were found in immune cells such as macrophages, a principle source of proinflammatory cytokines IL-1, IL-6, and TNF-*α* [19]. Although 5HT_4_ receptor mRNA was not detected in immune cells in animal studies [20], liberation of 5HT following activation of 5HT_4_ receptors could serve a critical role in infiltration and activation of macrophages via activation of their 5HT receptors, in intestinal inflammation [19, 21]. Due to the fact that increasing content of 5HT has demonstrated in UC, there may be an association between intestinal inflammation and a change in 5-HT content [18]. In addition in our previous study, we demonstrated that the blocking of 5HT_3_ receptors by ondansetron, a 5HT_3_ receptor antagonist, alleviated the colon injuries in experimental colitis [22]. Considering these data, the question arises as to whether the 5HT_4_ receptors like 5HT_3_ receptors are involved in pathogenesis of UC. If this hypothesis is true, it is likely that the activation of 5HT_4_ receptors deteriorates the severity of colitis. Therefore, our aim is to characterize the effect of administration of cisapride as a 5HT_4_ receptor agonist on an immune-based animal model of colitis (TNBS model) in rat. 

## 2. Methods

### 2.1. Animals 

Male Wistar rats (200 ± 20 g) obtained from the laboratory animal house of School of Pharmacy, Isfahan University of Medical Sciences were randomly distributed into several experimental groups. Animal quarters maintained a constant temperature (22 ± 1°C), relative humidity (55 ± 10%), and a 12 h light-dark cycle. Rats fed standard pelleted chow and water *ad libitum*. Animals were treated in accordance with the Guide for the Care and Use of Laboratory Animals as adopted and promulgated by the Animal Care Committee of the Isfahan University of Medical Sciences. 

### 2.2. Chemicals

Dexamethasone and cisapride were obtained from Iran Hormone Pharmaceutical Co. (Tehran, Iran) and Sigma Chemical Company (St. Louis, MO, USA), respectively. TNBS, hexadecyl trimethyl-ammonium bromide (HTAB), aprotinin A, bovine serum albumin, phenylmethylsulfonyl fluoride, benzethonium chloride, ethylene diamine tetra acetic acid (EDTA), and tween 20 were provided from Sigma Chemical Company (St. Louis, MO, USA). TNF-*α* (ALPCO, USA), IL-1*β* (ALPCO, USA), and IL-6 (ALPCO, USA) kits were used in order to analyze the biochemical variables.

### 2.3. Grouping

Rats were randomly assigned to 8 groups (6 animals in each) as follows. (Ia) TNBS-control group: rats received normal saline intraperitoneally (i.p) 2 hours following induction of colitis; (Ib) TNBS-control group: rats received normal saline orally (p.o) 2 hours following induction of colitis (IIa) normal group: cannulation was accomplished without induction of colitis (normal saline was administered instead of TNBS), and rats received normal saline i.p; (IIb) normal group: cannulation was accomplished without induction of colitis (normal saline was administered instead of TNBS), and rats received normal saline p.o; (IIIa) dexamethasone, i.p group: dexamethasone (1 mg/kg, i.p) was given 2 hours following induction of colitis [23]; (IIIb) dexamethasone, p.o group: dexamethasone (2 mg/kg, p.o) was given 2 hours following induction of colitis [23]; (IVa) cisapride, i.p group: cisapride (2 mg/kg, i.p) was administered 2 hours following induction of colitis [24]; (V) cisapride, p.o. group: cisapride (4 mg/kg, p.o) was administered 2 hours following induction of colitis [25].

### 2.4. Induction of Colitis

Colonic inflammation was induced according to the method described by Morris et al. [26]. Animals were fasted for 36 h and slightly anesthetized with diethyl ether. Rats were thereafter positioned on their right side, and a single intracolonic dose of 10 mg (50 mg/kg) TNBS dissolved in 0.25 mL of 50% ethanol (v/v) was administered through a polyethylene catheter inserted 8 cm proximal to the anus. It was followed by maintaining rats in a head down position for 2-3 minutes to avoid immediate anal leakage of the instillate, and thereafter the rats were returned to their cages with access to food and water *ad libitum*. Normal groups were given an enema of 0.25 mL of normal saline.

### 2.5. Measurement of Body Weight Changes and Diarrheal Status

Animal body weights and occurrence of diarrhoea were recorded daily throughout all the experiments. Percent of body weight loss was thereafter measured. Using arbitrary criteria (1) formed stools, (2) loosed stools, (3) diarrhea, fecal output was assessed daily for 6 days.

### 2.6. Macroscopic Studies

Animals were sacrificed by means of ether inhalation on day 6. For each animal, the distal colon was removed and cut longitudinally, slightly cleaned in physiological saline to remove fecal residues, weighed and processed for assessment by macroscopic, histological scores and biochemical markers. For each specimen, distal colon wet weight (mg) (8 cm from the anus) and weight/length ratio (mg/cm) were measured. Using scoring system depicted in Table 1 according to the criteria of Ballester et al., with slight modifications, the severity of macroscopically visible colonic damage was scored [27]. Pieces of damaged colon thereafter were collected and immediately frozen in liquid nitrogen for measurement of biochemical parameters. After taking photos from distal colons, ulcer area and percent of necrosis were determined according to our previously described method [15].

### 2.7. Histological Studies

Cross-sections were selected and embedded in paraffin. Full-thickness sections of 4 *μ*m were obtained at different levels and stained with haematoxylin and eosin (H&E). The histological damage was evaluated by a coworker pathologist, who was blinded to the study, according to the criteria previously described by Cooper et al. and Dieleman et al. [28, 29]. Total colitis index was then derived by summing 3 subscores (inflammation severity, inflammation extent, and crypt damage) on H&E-stained and coded sections. 

### 2.8. MPO Activity Determination

Myeloperoxidase (MPO) activity was measured according to the technique described by Bradley et al. [30] with some modification. Each segment was weighed and chopped in 1 mL of 50 mM potassium phosphate buffer involving 0.5% HTAB. Having chopped, we placed tissue in a homogenizing tube. The container was then rinsed with 2 × 1 mL HTAB in buffer solution. Afterwards, we added more buffer in order to have a concentration which was equivalent to 5 mL per 0.1 g of colon tissue and homogenized (15,000 rpm) for 4 × 45 s at 1 min intervals. The homogenate was placed in a sample tube, sonicated in an ice bath for 10 s, subjected to 3 cycles of freezing and thawing, and sonicated again for 10 s. The suspensions were centrifuged (15,000 rpm for 15 min in 4°C). The supernatant thereafter decanted for assessment. The MPO activity was analyzed spectrophotometrically as follows: 0.1 mL of the supernatant was added to 2.9 mL of 50 mM K_3_PO_4_ buffer (pH = 6.0) involving O-dianisidinedihydrochloride (0.167 mg/mL) and 0.005% hydrogen peroxide. The absorbance of the reaction mixture was recorded at a wave length of 450 nm by means of a UV-Vis spectrophotometer. The results are expressed as the change in absorbance/min/mg colonic wet weight.

### 2.9. Cytokine Assays

The amount of Rat TNF-*α*, IL-1*β*, and IL-6 in the colonic samples was quantified by commercially available enzyme-linked immunosorbent assay kits (ALPCO, USA) as described earlier [31].

## 3. Statistical Analysis

All data are expressed as mean ± S.E.M. Clinical activity score of colitis and macroscopic and histological scores were statistically analyzed using the Mann-Whitney *U* test. Differences in parametric data were determined by one-way analysis of variance (ANOVA) with TUKEY as post hoc test. Differences were considered statistically significant with *P* < 0.05.

## 4. Results

### 4.1. Changes in Rats' Body Weight and Diarrheal Status

TNBS- and cisapride-treated rats (i.p, p.o) showed loss of body weight after 6 days (*P* < 0.001), and there was no significant difference between these groups in aforementioned parameter. Animals in dexamethasone-treated groups (i.p, p.o) also experienced a significant loss of body weight in comparison with normal group; however, percent of body weight loss in these groups was significantly lower than TNBS-control or cisapride groups after 6 days (*P* < 0.05) (Table 2). Furthermore, there were no significant differences between i.p and p.o of either medication.

**Figure 1:**
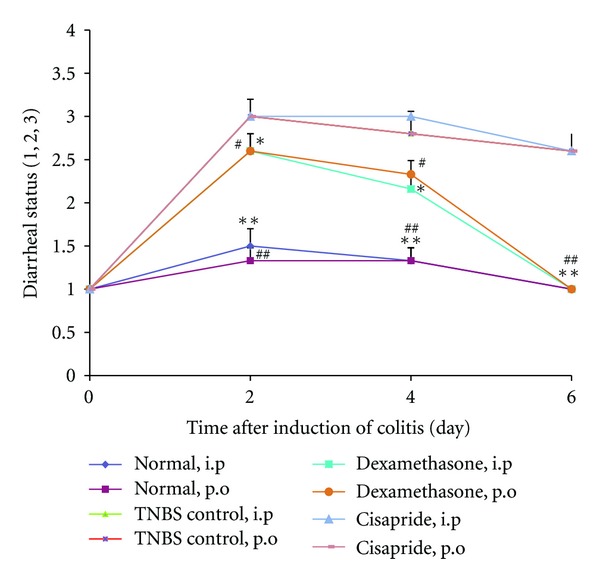
Changes in diarrheal status before (day 0) and during 6 days of treatment after induction of colitis (TNBS, 50 mg/kg) in rats. Values are means ± SEM (*n* = 6). **P < 0.01 and *P < 0.05 compared with TNBS-control group, i.p; ^##^ *P* < 0.01 and ^#^ *P* < 0.05 compared with TNBS-control group, p.o TNBS, 2, 4, 6-trinitrobenzenesulfonic acid. Stool consistency was daily checked in rats (1) formed stools, (2) loosed stools, and (3) diarrhea.

As can be noted in Figure 1, diarrheal status in TNBS- and cisapride-treated rats during all 6 days of the experiment was significantly high, in comparison to normal groups (*P* < 0.01). No significant difference was observed in the daily diarrheal status between animals treated with cisapride and TNBS over the experiment.

 Additionally, rats treated with dexamethasone exhibited a significant decrease in the diarrhea index especially after the initial 2 days of treatment subsequent to induction of colitis, in comparison with TNBS-control group (at least *P* < 0.05). 

### 4.2. Effect of Cisapride on Macroscopic Features

**Figure 2:**
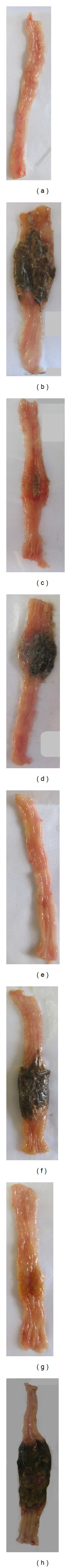
Macroscopic presentation of TNBS-induced colitis in rats. (a) normal rat, i.p; (b)TNBS-control rat, i.p (50 mg/kg); (c) dexamethasone-treated rat, i.p (1 mg/kg); (d) cisapride-treated rat, i.p (2 mg/kg); (e) normal rat, p.o; (f) TNBS-control rat, p.o (50 mg/kg); (g) dexamethasone-treated rat, p.o (2 mg/kg); (h) cisapride-treated rat, p.o (4 mg/kg). Note the extensive hyperemia, edema, ulceration, visible enlargement, and necrosis of the colon in B, D, F, and H groups. In C and G groups the damage score of colon was significantly decreased.

TNBS-control and cisapride groups (i.p, p.o) experienced severe inflammation, hemorrhage, ulcer, necrosis, and thickened colon wall 6 days after induction of colitis, while the normal macroscopic features were evident in colons of normal group (Table 2, Figure 2). There was also no significant difference in these variables between TNBS and cisapride-treated rats. Compared with TNBS-control group, there was a significant decrease in ulcer severity, weight/length ratio, ulcer area, and percent of necrosis in dexamethasone-(i.p, p.o) treated groups (*P* < 0.01). There were no significant differences between i.p and p.o of either medication.

### 4.3. Effect of Cisapride on Histopathological Features

**Figure 3:**
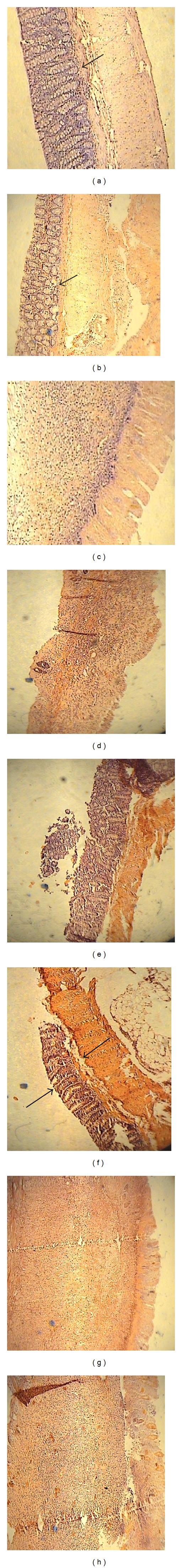
Microscopic presentation of TNBS-induced colitis in rats (hematoxylin and eosin staining; original magnification × 10). (a and b) Normal groups: mucus layer and crypts are normal; (c and d) TNBS-control groups: epithelial distortion, crypt damage, and inflammatory cell infiltrates; (e and f) dexamethasone (i.p and p.o, resp.): moderate mucosal and submucosal inflammation and mucosal inflammatory cell infiltrates; (g and h): cisapride (i.p and p.o, resp.) infiltration of neutrophils and destruction of mucosal architecture.

Figure 3 depicts histological assessment in studied groups, 6 days subsequent to induction of colitis. Normal group showed a normal architecture with intact epithelium in colonic mucosa. There were severe and intense transmural inflammation and/or diffuse necrosis, inflammatory granulomas, and submucosal neutrophils infiltration in TNBS-control (50 mg/kg) and cisapride (i.p, p.o) group. There was no significant difference in microscopic features between cisapride-treated and TNBS-control groups.

 Dexamethasone-treated groups (i.p, p.o) significantly showed less histopathological damages. These treatments reduced total colitis index (inflammation severity, inflammation extent, and crypt damage) in injurious colons (Table 2). Furthermore, these groups experienced reepithelization of the mucosal layer and reduced inflammatory cell infiltration in lamina propria. There were no significant differences between i.p and p.o of either medication.

### 4.4. Effect of Cisapride on Myeloperoxidase Activity

As can be shown in Table 3, MPO activity, a marker for leukocyte infiltration into the inflamed tissue, was markedly enhanced in the inflamed colons subsequent to the intrarectal TNBS administration versus normal groups. This result confirmed, the histological assessment which showed increased leucocyte infiltration in TNBS-control groups. Administration of dexamethasone (i.p, p.o) significantly declined the MPO activity level (*P* < 0.01). There were no significant differences between i.p and p.o of either medication or no differences between cisapride and TNBS administered with the same route.

### 4.5. Effect of Cisapride on Cytokines Profile

As can be noted in Table 3, TNF-*α*, IL-1*β*, and IL-6 contents soared in the TNBS-control groups, as compared with those of normal rats. Cisapride-treated groups (i.p, p.o) did not experience any significant changes in the levels of inflammatory cytokines compared with TNBS-control groups. These parameters were significantly lowered in rats treated with dexamethasone (i.p, p.o) (Table 3). In addition, there were no significant differences between i.p and p.o of either medication.

## 5. Discussion

 The present study assessed the effects of administration of cisapride on the severity of experimental colitis in rats. Cisapride-treated rats showed a colitis which was comparable to that of TNBS-control group. In fact, evaluation of clinical, macroscopic, histopathologic, and biochemical parameters showed that there was no significant difference between cisapride and TNBS-control groups. 

A suitable animal model of IBD is to show all the characteristics of a typical human IBD such as macroscopic, histopathological, and biochemical alternations accompanied by covering acute and chronic symptoms. Of the different models of induction of colitis, TNBS-induced colitis is one of the best which can mimic efficiently the pattern of inflammation similar to human ulcerative colitis. This model creates a simple process, reproducible colonic damage, and long-lasting damage accompanied by inflammatory cell infiltration and ulcers. Taking into account the fact that serotonin increase has been demonstrated in TNBS-induced colitis, this model is likely to be beneficial and suitable for analyzing the drugs which impact serotonin pathway in intestinal inflammation [15, 16, 32]. An increase was found in the bioavailability of 5-HT from mucosal epithelial cells in TNBS-induced colitis [16]. In addition, TNBS model could activate both Th_1_ and Th_2_ responses [33]. It can also induce both acute and chronic phases of colitis predicated upon experiment period [34]. Therefore, present study set out as a six-day experiment to assess the influence of chronic administration of cisapride on TNBS-induced colitis. The time period adopted in the present experiment was similar to the previous studies carried out on model of TNBS-induced chronic inflammation in rats [26]. 

An increase in the number of EC cells and 5-HT content was shown to be involved in intestinal mucosal inflammation such as ulcerative colitis [18]. It should be mentioned that serotoninergic receptors were found in immune cells including macrophages which are principle source of proinflammatory cytokines IL-1, IL-6, and TNF [19]. On the grounds of strategic location of EC, it can be inferred that 5-HT plays a part in infiltration and activation of macrophages in intestinal inflammation. Serotonin is also secreted by immune cells; hence, this exogenously added serotonin increases T-cell proliferation [19]. T cells are obviously involved in pathogenesis of IBD. Not only does Th_1_ cell activation liberate proinflammatory cytokines, but also it stimulates tissue macrophages in order to release additional proinflammatory cytokines (e.g., TNF-*α*, IL-1*β*, IL-6, IL-8, and IL-12), nitric oxide, and reactive oxygen species [35]. Amongst the intestinal immunomodulatory factors, cytokines are believed to serve a crucial role. To clarify, an imbalance between proinflammatory and anti-inflammatory cytokines is associated with the IBD pathogenesis. Proinflammatory cytokines (e.g., TNF-*α*, IL-1*β*, and IL-6) liberated from macrophages, neutrophils, and endothelial cells were shown to be overproduced in TNBS-induced colitis and in human IBD [36]. It should be noted that they can liberate other cytokines, arachidonic acid metabolites, and lytic enzymes by intestinal macrophages, neutrophils, smooth muscle cells, fibroblasts, and epithelial cells and thus result in edema, fibrosis, and necrosis [37]. Furthermore, TNF-*α* and IL-1*β* are the main mediators pertaining to neutrophil activation and mobilization [37]. In the present study, we found that the level of these proinflammatory cytokines increased subsequent to TNBS instillation. In addition, our findings reported that the colonic levels of these mediators are still high in cisapride-treated rats. This result is possibly due to stimulation of synthesis and/or release of these mediators. 

MPO activity has been used as a quantitative index of neutrophil influx into inflamed intestinal tissue [36]. In the preset study, MPO activity was conspicuously enhanced in TNBS-treated rats, and there was no significant difference in MPO activity between TNBS-control and cisapride groups. This high level of MPO activity and infiltration of inflammatory cells into the colonic tissue is consistent with severe macroscopic and microscopic damage scores in these groups. 

Most 5-HT is released from EC cells, and therefore the majority accumulation of 5HT is in GI tract. In addition, it has been demonstrated that an increase in the number of EC cells and in 5-HT content is associated with intestinal mucosal inflammation such as ulcerative colitis [18]. It is also worth nothing that amongst the variety of serotoninergic receptors, 5HT_4_ receptors exert extensive distribution in GI. In fact the highest expression of 5HT_4_ receptors is in the distal colon [17].

Based on the probable association between 5HT and intestinal inflammation and also the extensive distribution of 5HT_4_ receptors in colon, it can be inferred that this class of receptor maybe has a fundamental role in the pathogenesis of UC. It would be expected following the administration of cisapride as a 5HT_4_ receptor agonist, liberation of 5HT, and activation of 5HT receptors on macrophages that the severity of experimental TNBS-induced colitis would be aggravated. Our results showed that the severity of colitis in TNBS-control group, and cisapride-treated rats was similar and cisapride did not deteriorate the severity of experimental colitis in rat. This is likely to arise from the severity of colitis that reached its peak about 6 days following colitis induction, and no more severity can stem from cisapride which is a potent 5HT_4_ receptor agonist. On the other hand, we must notice that cisapride dosage was selected on the basis of previous studies using this drug to analyze the role of 5-HT_4_ receptors in GI function in rat, and amongst these employed doses we selected the high dose of cisapride in our study. Therefore, it seems that dose of cisapride would not be a probable limitation in this work. Furthermore, it can be deduced that because the severity of colitis produced by TNBS is massive (through various pathways), it is likely that cisapride could not bring about more colitis damages through 5HT_4_ receptors. In fact for determination of the role of 5HT_4_ receptor in the pathogenesis of colitis, alongside the assessment of the effect of 5HT_4_ receptor activation, it should be better to investigate the effect of blocking of this receptor on the severity of experimental colitis. Therefore we suggest further researches using administration of one 5HT_4_ antagonist and evaluation of its effects on severity of experimental colitis. If the administration of a 5HT_4_ receptor antagonist could ameliorate the colonic injuries and also cisapride could antagonize the probable beneficial effect of 5HT_4_ receptor antagonist, the important role of 5HT_4_ receptors in the pathogenesis of colitis will be revealed. 

 In conclusion, administration of cisapride, a 5HT_4_ receptor agonist, does not aggravate the severity of colonic injuries in TNBS-induced colitis in rat. Further studies are required using administration of 5HT_4_ receptor agonist/antagonist to investigate the exact role of 5HT_4_ receptors in the pathogenesis of ulcerative colitis.

## Figures and Tables

**Table 1:** Scoring criteria for assessment of macroscopic rat colonic injuries.

Adhesions	0 No adhesions
	1 Difficult dissection
	2 Visible adhesions
	3 “Wrapped” intestine

Obstruction	0 No obstruction
	1 Need for gentle manual cleaning
	2 Fecal impaction

Thickening	0 Similar to uninflamed intestine
	1 Thicker than normal (~1-2 mm)
	2 Much thicker than normal (>2 mm)

Hyperemia	0 Similar to uninflamed intestine
	1 Mild and generalized or intense but localized hyperemia
	2 Intense and localized hyperemia
	3 Frank hemorrhage

Necrosis	0 No signs of necrosis
	1 Small areas of necrosis
	2 Patchy necrosis
	3 Focal necrosis <0.8 cm
	4 Focal necrosis >0.8 cm
	5 Extended necrotic lesion

**Table 2:** Effect of cisapride (2 mg/kg, i.p; 4 mg/kg, p.o: daily) on macroscopic and histopathological parameters of the rat colon 6 days after induction of colitis with TNBS (50 mg/kg).

Group	Colonic weight/length ratio (mg/cm)	Body weight loss after 6 days (%)	Ulcer severity (0–15)	Total colitis index (0–10)	Ulcer area (cm^2^)	Necrosis (%)
Normal (i.p)	64.8 ± 2.3	−1.9 ± 0.6	0.0	0.0	0.0	0.0
Normal (p.o)	72.1 ± 2.8	−1.6 ± 0.5	0.0	0.0	0.0	0.0
TNBS control (i.p)	256.3 ± 9.5	7.7 ± 0.7	12.2 ± 0.6	9.9 ± 0.1	6.2 ± 0.1	51.5 ± 2.8
TNBS control (p.o)	252.1 ± 9.4	8.2 ± 0.8	11.8 ± 0.7	9.9 ± 0.1	6.0 ± 0.2	48.8 ± 3.8
Cis. (2 mg/kg, i.p)	256.3 ± 7.1	7.1 ± 0.6	12.0 ± 0.5	9.8 ± 0.1	5.7 ± 0.3	48.2 ± 3.5
Cis (4 mg/kg, p.o)	253.1 ± 8.2	8.0 ± 0.9	12.2 ± 0.5	9.5 ± 0.2	6.0 ± 0.3	50.0 ± 1.8
Dex. (1 mg/kg, i.p)	161.9 ± 27.4^∗∗^	3.9 ± 0.3^∗^	5.33 ± 1.4^∗∗^	4.8 ± 0.8^∗∗^	4.0 ± 0.7^∗∗^	27.0 ± 7.9^∗∗^
Dex. (2 mg/kg, p.o)	163.5 ± 22.9^##^	4.7 ± 0.8^#^	4.7 ± 1.4^##^	4.9 ± 0.5^##^	3.9 ± 0.3^##^	22.2 ± 5.2^##^

TNBS, 2, 4, 6-trinitrobenzenesulfonic acid, cis: cisapride, dex.: dexamethasone, i.p: intraperitoneal, p.o: oral.

Values are means ± SEM: *n* = 6.

**P < 0.01 and *P < 0.05: significant difference compared to TNBS-control group, i.p; ^##^ *P* < 0.01 and ^#^ *P* < 0.05: significant difference compared to TNBS-control group, p.o.

**Table 3:** Biochemical parameters of the rat colon 6 days after induction of colitis with TNBS (50 mg/kg).

Group	MPO activity	TNF-*α*	IL-6	IL-1*β*
	(unit/100 mg wet tissue)	(pg/g wet tissue)	(pg/g wet tissue)	(pg/g wet tissue)
Normal (i.p)	0.6 ± 0.1	141.0 ± 13.8	3663.3 ± 240.2	1647.5 ± 452.4
Normal (p.o)	0.6 ± 0.1	144.6 ± 12.7	3646.7 ± 261.0	1768.7 ± 578.8
TNBS control (i.p)	3.4 ± 0.4	249.9 ± 26.6	5210.3 ± 457.9	15296.0 ± 1579.7
TNBS control (p.o)	3.4 ± 0.4	251.8 ± 18.5	5272.0 ± 364.5	15563.4 ± 1476.5
Cis (2 mg/kg, i.p)	3.5 ± 0.3	254.1 ± 21.5	5133.7 ± 352.2	15378.3 ±1252.5
Cis (4 mg/kg, p.o)	3.6 ± 0.2	251.6 ± 15.6	5224.8 ± 263.9	15825.3 ± 1198.2
Dex. (1 mg/kg, i.p)	1.9 ± 0.4^∗∗^	160.6 ±12.6^∗^	3821.7 ± 270.7^∗^	8641.2 ± 985.2^∗∗^
Dex. (2 mg/kg, p.o)	1.8 ± 0.1^##^	164.7 ± 10.1^#^	3870.7 ± 240.2^#^	9162.7 ± 1574.4^#^

TNBS: 2, 4, 6-trinitrobenzenesulfonic acid, cis: cisapride, dex.: dexamethasone, i.p: intraperitoneal, p.o: oral.

Values are means ± SEM: *n* = 6.

**P < 0.01 and *P < 0.05: significant difference compared to TNBS-control group, i.p; ^##^ *P* < 0.01 and ^#^ *P* < 0.05: significant difference compared to TNBS-control group, p.o.
